# Plantar-flexor Static Stretch Training Effect on Eccentric and Concentric Peak Torque – A comparative Study of Trained versus Untrained Subjects

**DOI:** 10.2478/v10078-012-0063-z

**Published:** 2012-10-23

**Authors:** Amr Almaz Abdel-aziem, Walaa Sayed Mohammad

**Affiliations:** 1 Department of Biomechanics, Faculty of Physical Therapy, Cairo University, Egypt.

**Keywords:** calf muscle, isokinetic torque, static stretching

## Abstract

The aim of this study was to examine the long-term effects of static stretching of the plantar-flexor muscles on eccentric and concentric torque and ankle dorsiflexion range of motion in healthy subjects. Seventy five healthy male volunteers, with no previous history of trauma to the calf that required surgery, absence of knee flexion contracture and no history of neurologic dysfunction or disease, systemic disease affecting the lower extremities were selected for this study. The participants were divided into three equal groups. The control group did not stretch the plantar-flexor muscles. Two Experimental groups (trained and untrained) were instructed to perform static stretching exercise of 30 second duration and 5 repetitions twice daily. The stretching sessions were carried out 5 days a week for 6 weeks. The dorsiflexion range of motion was measured in all subjects. Also measured was the eccentric and concentric torque of plantar-flexors at angular velocities of 30 and 120°/s pre and post stretching. Analysis of variance showed a significant increase in plantar-flexor eccentric and concentric torque (p < 0.05) of trained and untrained groups, and an increase in dorsiflexion range of motion (p < 0.05) at both angular velocities for the untrained group only. The static stretching program of plantar-flexors was effective in increasing the concentric and eccentric plantarflexion torque at angular velocities of 30 and 120°/s. Increases in plantar-flexors flexibility were observed in untrained subjects.

## Introduction

Strength and flexibility are common components of exercise programs; however, it is not clear how best include both of these elements in a single training program. Training for flexibility and strength is widely recommended for those who wish to attain good fitness levels and a better quality of life that is gained by preventing muscle injury and soreness or even enhancing performance. Many activities rely on strength, but strength performance may be diminished by stretching. Therefore, it is important to understand this phenomenon when prescribing physical exercise programs (Rubini et al., 2007).

Many authors have studied the acute effect of a stretching routines on strength performance, but the results are often controversial. Various studies found that stretching exercises preceding the main strength activity significantly decreased performance (Avela et al., 2004; Behm et al., 2006; Brandenburg, 2006; Cramer et al., 2005; Derek et al., 2005; Marek et al., 2005; Nelson et al., 2001; Nelson et al., 2005; Power et al., 2004; Rubini et al., 2005; Yamaguchi et al., 2006). These studies used stretching exercise of lower extremities and found decreases in strength ranging from 4.5 to 28%, irrespective to the testing mode (i.e. isometric, isotonic or isokinetic) (Rubini et al., 2007).

Very few studies have looked into the chronic effects of stretching on strength performance. Worrell et al. (1994) used static stretching and proprioceptive neuromuscular facilitation (PNF) ‘contract-relax’ methods to train the flexibility of the hamstrings. Exercises were performed five times a week, for 3 consecutive weeks, with 20 minute sessions. The study showed there was no significant gains in flexibility, but a 8.5 and 13.5% increases in eccentric peak torque measured at 60 and 120°/s, respectively, and a 11.2% increase in concentric peak torque at 120°/sec. Handel et al. (1997) used the PNF ‘contract-relax’ method to train the knee extensor and flexor muscles. Exercises were performed three times a week for 8 consecutive weeks, with a total of 86 minutes 40 seconds in each session. The study indicated that there was a significant increase in flexibility (up to 6.3%). In the knee flexor and extensor muscles eccentric peak torque increased by 18.2 and 23.0%, respectively, while the knee flexor concentric peak torque and knee flexor isometric peak torque results improved by 9.4 and 11.3%.

During an eccentric contraction, mechanical work is absorbed by the series elastic component of the muscle as potential energy, which is used during the immediate concentric contraction (Cavagna, 1970; Cavagna et al., 1968). This condition of eccentric contraction followed by concentric contraction occurs during gait and running. For example, the quadriceps femoris undergoes an eccentric contraction during heel strike and concentric contraction at push-off. The same is true for the gastrocnemius and soleus (plantar-flexors) muscles during midstance and push-off. The speed of the eccentric contraction and muscle length are the factors that determine the amount of energy absorbed by muscles (Cavagna, 1970). Thus, if the length of the muscle can be increased, more forces will be absorbed during the eccentric contraction and increased force will be generated during the concentric contraction. Worrell et al. (1994) stated that patients with lower extremity overuse injuries would benefit by muscle stretching because greater force will be absorbed, lessening the overload on weakened and inflamed tissues. In addition, theoretically, muscle performance will be increased for activities of daily living or sports by increasing the potential energy available for concentric contractions.

There is contradictory data on the effect of stretching exercises on flexibility of plantar-flexors. Therefore, the purpose of this study was 1) to examine the effect of static stretching on plantar-flexor flexibility and 2) to determine the long-term effect of static stretching on concentric and eccentric peak torque of plantar-flexors of trained and untrained subjects.

## Material and Methods

### Subjects

Seventy five volunteers from physical therapy students and staff participated in this study. Inclusion criteria for this study were as follows: (1) no block at the talocrural joint that would limit ankle dorsiflexion or plantar flexion, and no limitation of subtalar joint mobility that would limit foot inversion or eversion motion; (2) no previous history of trauma to the calf that required surgery; (3) absence of knee flexion contracture; and (4) no history of neurologic dysfunction or disease, systemic disease affecting the lower extremities or ambulation, (5) no macrotrauma involving bone or nerve injury to the lower extremity.

The subjects were divided into three equal groups; the first group (untrained group; age = 22.3 ± 2.3 years, body height 171 ± 4.7 cm, body mass = 68.2 ± 6.4 kg). The second group (trained group; age = 22.7 ± 2.7 years, body height 170 ± 6.3 cm, body mass = 68.5 ± 5.6 kg). Both groups performed static stretching exercises. The third group (untrained group; age = 21.9 ± 4.1 years, body height 169 ± 5.9 cm, body mass = 66.2 ± 7.4 kg) served as a control group and did not stretch. A subject was considered trained if they engaged in an aerobic activity at least three times weekly for more than 20 minutes a session (Seymour and Bacharach, 1990). Informed consent was obtained from all subjects participating in this study before any measurements were taken. The research was approved by the ethics committee of the Faculty of Physical Therapy, Cairo University.

### Instrumentation

A universal goniometer (UG) with a double-armed full-circle protractor made of transparent plastic (Bench-mark Medical, Inc) was used to measure ankle dorsiflexion range of motion (ROM). The length of the arms was 20.3 cm (8 in) and the scale of the protractor was marked in 1° increments. The UG was selected because it is commonly used by physical therapists when making measurements of joint mobility.

Isokinetic dynamometer (Biodex Multi-joint System 3) was used to measure the eccentric and concentric torque of calf muscles before (Pre) and after six weeks of static stretching (Post) of plantar-flexors. The test protocol was supplied by the manufacturer and was strictly followed. Prior to data collection, the Biodex device was calibrated, the testing procedures were explained, and each subject was positioned.

### Flexibility assessment

Each volunteer lay prone on a standard treatment table. Maximum active ankle dorsiflexion ROM of the right ankle was measured with the knee straight. This position was selected because lying prone is a functional position for assessment of active ankle dorsiflexion ROM. Both hip and knee joints are extended simultaneously, simulating the stance phase (of the gait cycle) just before heel-off, where the greatest demand for active ankle dorsiflexion ROM, and gastrocnemius is maximally stretched by a combination of knee extension and ankle dorsiflexion (Stauffer et al., 1977). The right hip was placed in neutral rotation with the knee in terminal extension and the foot hanging over the table’s edge to permit full active ankle dorsiflexion ROM. The examiner sat in a standard chair at the level of the subject’s right leg-ankle-foot complex. Each subject was requested to actively dorsiflex and plantar flex the right ankle joint through the available ROM 4 times for the purpose of preconditioning the soft tissues of the calf muscle-tenden unit (MTU). This maneuver was recommended by Zito et al. (1997) because repeated stretch cycles before the testing procedure improves the reproducibility of a measurement, controlling for temporary lengthening mechanisms associated with stretching of connective tissues. The examiner observed the subject’s ankle motion closely to prevent foot eversion during maximum active ankle dorsiflexion ROM, because this extraneous movement would involve dorsiflexion of midtarsal and talocrural joints. The active ankle dorsiflexion ROM was used because the volitional movement of the dorsiflexor muscles would be expected to inhibit the calf MTU via reciprocal inhibition (Kandel et al., 2000).

Once the terminal position of active ankle dorsiflexion ROM was achieved after the 4 repetitions, the examiner measured the amount of ankle dorsiflexion using a technique described by Valmassy (1996). A right angle formed by the intersection of the leg’s long axis with the foot’s long axis was assumed to be the starting position of ankle joint sagittal plane motion. The universal goniometer’s stationary arm was aligned parallel with the fibular head, whereas the moveable arm followed the foot’s plantar surface just inferior to the fifth metatarsal. The examiner read the measurement scale and reported the results.

After the baseline measurement was obtained, the subjects were instructed to hold the stretch for 30 seconds and repeat it 4 times (total of 5 repetitions) with 10-second rest intervals between repetitions. The participants were instructed to perform the stretching exercise twice daily, with at least 4 hours between sessions, for a total ten 30-second stretches per day. The stretching sessions were carried out 5 days a week for 6 weeks because this frequency exceeded that reported by most of the previous investigators (Bohannon et al., 1994; Grady and Saxena, 1991; Muir et al., 1999) who did not find lasting gains in active ankle dorsiflexion ROM, also exceeded the stretching technique conducted by Johanson et al. (2008) who proved that dorsiflexion ROM increased after a gastrocnemius stretching program.

### Stretching protocol

To stretch the plantar-flexor muscles, subjects stood barefoot about 2 to 3 feet from a solid wall. While facing the wall with their right foot perpendicular to it (Kisner and Colby, 1966), they were instructed to move the right foot backward while keeping the left foot forward, placing their hands against the wall and maintaining their right hip and knee in extension with the foot kept flat on the floor. This posture simulates the position of the ankle joint of the posterior leg during the stance phase of gait just before heel rise (Stauffer et al., 1977). No attempt to keep the subject’s subtalar joint in a neutral position as Worrell et al. (1994) reported no difference in active ankle dorsiflexion ROM between the supinated and pronated stretching positions. Subjects in our study were instructed to move their right foot back from the wall until they felt a substantial pull in the posterior calf that was just short of being painful. They were asked to maintain this sensation throughout the session either by leaning further into the wall or by moving the right foot even farther back from the wall. The stretching sessions were to be performed between 10 a.m. and 5 p.m. The posttest was conducted on a minimum of 60 and a maximum of 72 hours between the last bout of stretching.

### Isokinetic testing

Each subject performed a warm-up for 5 min, on a stationary bicycle, pedaling at a comfortable pace of 60 – 70 revolutions per minute and 5 min of stretching exercises for plantarflexors and dorsiflexors. The participants were tested in plantar-flexion-dorsiflexion movements using the Biodex multi joint system 3-isokinetic dynamometer (Biodex Medical Systems, Inc, Shirley, NY). The angular velocities were 30 and 120°/s for plantarflexion and dorsiflexion movements. Each subject was seated on the Biodex chair and stabilized by straps, with the axis of Biodex dynamometer aligned with the lateral malleolus and the angle of hip joint at 80° flexion (0° neutral position). In the ankle plantarflexion-dorsiflexion test a knee pad was placed under distal femur and secured with a strap allowing for approximately 20° to 30° of knee flexion. Also, the examiner ensured that the subject’s lower leg was parallel to the floor to diminish the potential for dynamic hamstring activity contributing to the generated torque (Lentell et al., 1988).

**Figure 1:**
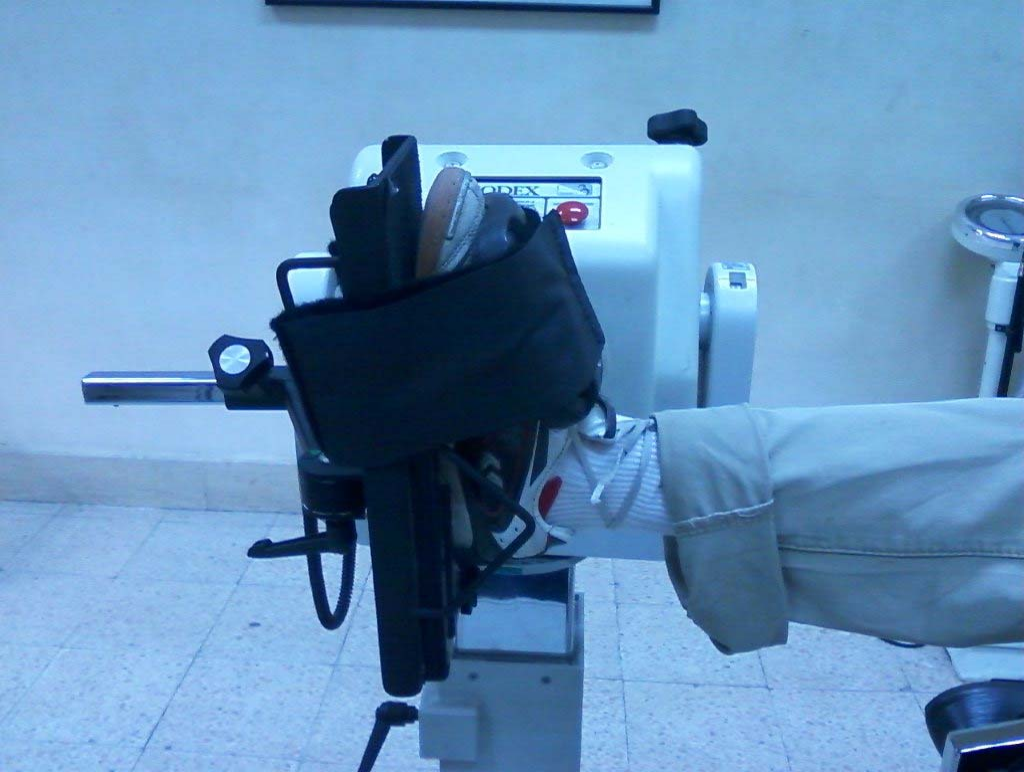
Foot position during measurement of plantar-flexor muscles eccentric and concentric peak torque

The foot and ankle were positioned into plantarflexion/dorsiflexion attachment with straps to secure the foot (Figure 1). Once positioned, the participant’s active range of motion was used to determine the start and stop angles. The subjects were also allowed to perform 10 submaximal contractions (familiarization trials) through the predetermined ROM at eccentric/concentric mode of testing with speeds of 30 and 120°/s to familiarize them with the dynamometer before the actual test. A rest period of 30 s was allowed between warm up and the actual test, and 1 min was allowed between testing speeds of 30 and 120°/s.

The testing protocol consisted of an eccentric loading of the plantar-flexors muscle group, followed by an immediate concentric plantar-flexors muscle contraction. In order to accomplish this, eccentric muscle contraction occurred during passive ankle dorsiflexion mode, and the concentric phase occurred during the ankle plantarflexion mode. Subjects received standardized verbal cues of “hold” during the eccentric phase and “pull” during the concentric phase, with instruction “not to relax between the two stages and maintain plantar-flexors contraction throughout the arc of procedures”. This procedure was repeated 5 times at the two angular velocities, the single highest value for each test was used for data analysis.

### Data analysis

Data was analysed using a Statistical Package for Social Sciences (SPSS) version 15.0. A multiple measures analysis of variance (MANOVA/mode of contraction and angular velocity) was used to compare plantar-flexor isokinetic values among control, trained and untrained (pre and post) stretching groups. Finally, one-way analysis of variance (ANOVA) was conducted to compare among the control (pre, post), trained and untrained (posttest and pretest) scores of the dorsiflexion ROM. The level of significance was set at 0,05 for all statistical tests with the least significant difference (LSD) used to locate the source of differences.

## Results

The peak torque values for the calf muscles during concentric and eccentric contraction at pre and post stretching for the three groups at 30 and 120°/s angular velocities are shown in Table 1. The peak torque values for the plantar-flexor muscles during concentric contraction was always lower than that during the eccentric mode at both angular velocities 30 and 120°/s. Regarding, the angular velocity, the peak torque values for the plantar-flexor muscles at 120°/s was always higher than that at 30°/s during eccentric mode of contraction. However, at 30°/s the peak torque value was higher after stretching of the calf muscles either during concentric or eccentric contractions.

During concentric contractions at angular velocities of 30 and 120°/s, there was no significant difference between pre and post peak torque values in the control group (*p* > 0.05). However, there was a significant difference between pre and post peak torque values in untrained groups at angular velocities of 30 and 120°/s (*p* < 0.05). Moreover, there was a significant difference between pre and post peak torque values of trained groups at angular velocities 30°/s and 120°/s (*p* < 0.05).

During eccentric contractions, there was no significant difference between pre and post peak torque values in the control group (*p* > 0.05). However, there was a significant difference between pre and post peak torque values in the untrained group at angular velocities of 30 and 120°/s (*p* < 0.05). Moreover, there was a significant difference between pre and post peak torque values in the trained groups at angular velocities of 30 and 120°/s (*p* < 0.05).

There was no significant increase in the dorsiflexion ROM in the control and trained groups. However, there was a significant increase in the dorsiflexion ROM in the untrained group (*p* < 0.05). The dorsiflexion ROM values of the three groups are shown in Table 2.

## Discussion

Static stretching is used extensively in physical therapy and rehabilitation programs. The results of this study proved that plantar-flexor muscles static stretching increased ankle dorsiflexion ROM of untrained group without consideration of the subtalar joint position. This finding concurs with the findings of Worrell et al. (1994) who reported an increase in ankle dorsiflexion ROM after gastrocnemius stretching with the subtalar joint positioned in either supination or pronation. The ROM of the trained group did not increase what may be due to regular exercise which increased ROM.

The improvement in the flexibility of plantar-flexor muscles of untrained group is not coincident with the results of previous investigators (Bohannon et al., 1994; Grady and Saxena, 1991; Muir et al., 1999) where no significant gains in active ankle dorsiflexion ROM were found. However, the results of the current study supported by findings of Johanson et al. (2008) proved that dorsiflexion ROM increased after a gastrocnemius stretching program. This may be due to the stretching sessions of our study which were carried out at least 5 days a week for 6 weeks. The frequency and duration exceeded those reported by all previously mentioned researchers.

The mechanism underlying the increase in joint ROM can be explained by the effect of stretching. In animal studies, the chronic effect of stretching clearly changed both the contractile (Tabary et al., 1972; Williams and Goldspink, 1973), and passive ‘non contractile’ (Warren et al., 1976) elements of skeletal muscle, but similar changes after static stretching in human muscle have not been demonstrated (Lieber, 2002). In contrast, researchers (Halbertsma et al., 1996; Magnusson et al., 1998; Magnusson et al., 1996) have shown that static stretching of the human hamstrings muscle increased joint ROM without a concomitant decrease in stiffness or electromyographic activity of the stretched hamstrings muscle. These findings suggested that a central, rather than a peripheral, mechanism causes the increase in joint ROM after static stretching, and increased tolerance to stretching is the proposed central mechanism (Halbertsma et al., 1996; Lieber, 2002; Magnusson et al., 1998; Magnusson et al., 1996). If increased tolerance to stretching resulted in increased ankle dorsiflexion range in the study participants, joint positioning may not have been as relevant as it would have been if mechanical changes occurred within the contractile or passive elements of the gastrocnemius muscle.

The results of this study indicated that all subjects produced significantly higher eccentric torque values at 120 than at 30°/s, and lower concentric torque values at 120 than at 30o/s. These results are consistent with previous studies (Duncan et al., 1989; Walmsley et al., 1986). The eccentric force-velocity relationship indicates that peak torque increases with velocity, unlike the concentric force-velocity relationship in which peak torque decreases as velocity increases, which may explain our results.

Moreover, the results revealed a significant increase in plantar-flexor peak torque concentrically and eccentrically at angular velocities of 30 and 120°/s for experimental groups (trained and untrained). The greater improvement of the peak torque experimental groups proved that changes are caused by static stretching and could not be attributed to other factors such as learning, or repeated testing.

The improvement in concentric peak torque production at angular velocities of 30 and 120°/s resulted from the increased storage of potential energy during eccentric loading, which is used in the immediate concentric contraction (Asmussen and Bonde-Petersen, 1974; Cavagna, 1970; Cavagna et al., 1986; Wilson et al., 1991). This added potential energy must be used instantaneously following the eccentric contraction (Pousson et al., 1990). During the evaluation procedure of the current study the eccentric testing was followed immediately by concentric testing. Svantesson et al. (1994) found that, if the eccentric contraction of the plantarflexor was followed by a concentric contraction, it will increase the values of the concentric contraction. Wilson et al. (1992) demonstrated that, by increasing pectoralis and deltoid flexibility, series elastic component stiffness was significantly reduced during a 70% maximal bench press repetition. Moreover, they reported that the initial concentric work of the bench press was significantly increased after stretching. In addition, the bench load increased by 5.4%, which was not significant.

Increases in eccentric torque production at angular velocities of 30 and 120°/s can be attributed to the increase in plantar-flexor muscle flexibility and increasing the compliance of the series elastic component that results in a greater ability to store potential energy (Asmussen and Bonde-Petersen, 1974; Cavagna, 1970; Cavagna et al., 1986; Wilson et al., 1992; Wilson et al., 1991). This result is supported by the findings of Worrell et al. (1994) who found that there was significant increase in eccentric torque of hamstrings atangular velocities of 60 and 120°/s post static stretch training.

Yamashita et al. (1993) reported that stretching of a musculotendinous unit may also affect neuromuscular transmission. They conducted a study on rats and proved that stretching a rat soleus muscle by 10 and 20% increased posttetanic potentiation of the miniature end-plate potential, which indicates increased Ca^2+^ conductance in the nerve terminal. This increase in intracellular free Ca^2+^ facilitates neurotransmitter release. Theoretically, muscle force generation should increase as a result of increased transmitter release. Therefore, the increase in the plantar-flexor concentric and eccentric torque of trained and untrained groups may be due in part to factors other than changes in series elastic component stiffness and flexibility.

Finally, chronic static stretching leads to increased muscle force concentrically and eccentrically which differs from its acute effect when applied just before athletic activities, which produce a reduction of muscle strength and power. So, it is advisable to apply static stretching as a part of regular training rather than performing it just before the physical activities which will produce an adverse impact on subjects performance.

### Limitations

A limitation of our study was the inclusion of only healthy individuals without limitation in ankle dorsiflexion ROM. In future research, scientists must investigate the effect of static stretching on concentric and eccentric plantar-flexor torque for subjects suffering from ankle dorsiflexion ROM limitations. During stretching, the position of subtalar joint either supinated or pronated was not observed or checked. Future researchers may compare the effect of ankle stretching in supination versus pronation on the plantar-flexor peak torque. Gender in this study was limited to males only. So, the appropriateness of generalizing the results is confined to this specific population. Finally, the plantar-flexors peak torque was measured in the open kinetic chain only in this study, so caution must be taken when generalizing these results to closed kinetic chain activities. Additional studies are needed to determine the effect of increasing plantar-flexors flexibility on closed kinetic chain activities.

## Conclusions

The results of this study revealed that using static stretching techniques 5 repetitions of 30 s/twice daily, five times per week for six weeks, produced a significant increase in plantar-flexors flexibility for untrained individuals regardless of the subtalar joint position. Moreover, static stretching increased the isokinetic eccentric and concentric plantar-flexor peak torque at angular velocities of 30 and 120°/sec for trained and untrained individuals, which is different than the acute effects of stretching, which reduces muscle strength and power.

## Figures and Tables

**Table 1:** The mean values of peak torque (±SD) for the calf muscles during concentric and eccentric modes of contraction at 30°/s and 120°/s

Variables		Control Pre	Control Post	Pre-trained	Post-trained	Pre untrained	Post-untrained
Plantar-flexor concentric torque (Nm)	30°/sec	78.76 ± 13.36	78.84 ± 13.16	82.28 ± 15.46	89.80 ± 14.31	80.16 ± 14.57	80.04 ± 14.22
	120°/sec	49.75 ± 6.94	50.00 ± 6.94	55.04 ± 10.77	61.35 ± 9.78	50.96 ± 7.64	58.42 ± 7.64
Plantar-flexor eccentric torque (Nm)	30°/sec	80.16 ± 13.61	81.04 ± 13.84	85.61 ± 14.99	95.81 ± 12.58	81.08 ± 12.98	90.79 ± 14.03
	120°/sec	87.58 ± 15.21	87.96 ± 15.17	92.52 ± 10.74	104.09 ± 10.45	88.47 ± 9.64	96.84 ± 11.95

**Table 2:** The mean values of pre- and post-stretching dorsiflexion ROM of the three groups

Groups	Control	Untrained	Trained
Pre dorsiflexion (ROM)	8.90 ± 2.09	8.38 ± 2.07	11.02 ± 2.56
Post dorsiflexion (ROM)	9.02 ± 1.86	14.34 ± 2.69	12.12 ± 2.68
